# Surface PEGylation of Mesoporous Silica Nanorods (MSNR): Effect on loading, release, and delivery of mitoxantrone in hypoxic cancer cells

**DOI:** 10.1038/s41598-017-02531-4

**Published:** 2017-05-23

**Authors:** Amit Wani, Galbokka H. Layan Savithra, Ayat Abyad, Shrey Kanvinde, Jing Li, Stephanie Brock, David Oupický

**Affiliations:** 1 Department of Pharmaceutical Sciences, Wayne State University, Detroit, MI 48201 USA; 2 Department of Chemistry, Wayne State University, Detroit, MI 48202 USA; 3 Department of Pharmaceutical Sciences, Center for Drug Delivery and Nanomedicine, University of Nebraska Medical Center, Omaha, NE 68198 United States

## Abstract

Mesoporous silica nanomaterials show great potential to deliver chemotherapeutics for cancer treatment. The key challenges in the development of injectable mesoporous silica formulations are colloidal instability, hemolysis and inefficient drug loading and release. In this study, we evaluated the effect of PEGylation of mesoporous silica nanorods (MSNR) on hemolysis, colloidal stability, mitoxantrone (MTX) loading, *in vitro* MTX release, and cellular MTX delivery under hypoxic conditions. We found that PEGylation prevented dose-dependent hemolysis in the concentrations studied (0–10 mg/ml) and improved colloidal stability of MSNR. A negative effect of PEGylation on MTX loading was observed but PEGylated MSNR (PMSNR) demonstrated increased MTX release compared to non-PEGylated particles. Under hypoxic conditions, a decrease in the IC50 of MTX and MTX-loaded MSNR was observed when compared to normoxic conditions. These results showed that MSNR could deliver the chemotherapeutic agent, MTX to tumor cells and induce effective cell killing. However, the effect of PEGylation needs to be carefully studied due to the observed adverse effect on drug loading.

## Introduction

The application of nanoparticles in anticancer drug delivery has attracted much attention in recent decades^1, 2^. Various nanoparticle-based drug delivery systems have been developed to deliver chemotherapeutic agents to overcome drug resistance^3^, to improve drug bioavailability^4^, and to achieve selective cellular targeting while diminishing side effects of chemotherapy^5^. Inorganic materials such as mesoporous silicas offer a great potential as drug delivery systems due to their high drug loading, tunable pore size and pore volume, control over shape of the particles, easy surface modifications, and excellent biocompatibility. While strong evidence documents that size has a dominant effect on the drug delivery performance of nanoparticles, particle shape has emerged as another important factor that can be exploited for fine-tuning the particle performance. It has been well established that shape of nanoparticles has significant impact on cellular uptake^6^. Further, Ghandehari *et al*. have shown that PEGylated gold nanorods had higher tumor accumulation than PEGylated gold nanospheres in orthotopic ovarian tumor xenograft in mice^7^. Rod shaped particles also showed increase in the total blood circulation time compared to spherical nanoparticles, confirming that shape is an important characteristic of nanoparticles in drug delivery^7, 8^. Mesoporous silica nanoparticles (MSN) have been used to deliver chemotherapeutic agents and nucleic acids *in vitro* ^9–11^. MSN can encapsulate and protect hydrophobic as well as hydrophilic molecules and allow for controlled drug delivery^12^.

*In vivo* application of MSN in cancer treatment has been investigated previously with some promising activity^12, 13^. However, colloidal instability^14^ and hemolysis^15^ were major drawbacks in the development of successful MSN drug delivery systems. Lu *et al*. addressed the problem of colloidal instability by surface modification of MSN with phosphonate groups to prevent aggregation of the particles by electrostatic stabilization^16^. Though such surface modifications improved colloidal stability, the issue of hemolysis was not satisfactorily addressed. PEGylation is a frequent strategy to improve colloidal stability by providing steric surface hindrance to improve particle dispersion and to decrease hemolysis^12, 15, 17^. Indeed, biodistribution studies of PEG-MSN showed longer blood circulation with significantly less phagocytosis in the liver and spleen and a decrease in the capture by the capillary vessel beds in the lung^18^. However, the effect of PEGylation on drug loading, drug release and *in vitro* cellular drug delivery using MSN remains largely unaddressed.

Mitoxantrone (MTX) is an anthraquinoline anticancer agent that has been extensively studied and used in the treatment of breast and prostate cancer^19^. MTX exerts antiproliferative activity in various cancer types by interfering with DNA synthesis through intercalation and stabilization of DNA topoisomerase II cleavable complex^20^. Cardiotoxicity, a severe side effect of anthraquinoline derivatives, may be overcome by localizing drug at the tumor site through a nanomedicine approach^21^. Although MTX-loaded solid lipid nanoparticles^22^, PLGA nanoparticles and liposomes^23^ have been developed, low loading capacity and uncontrolled MTX release prevented their use in preclinical applications. Shi *et al*. reported that mesoporous silica gives more control over the loading capacity and release profile of weakly basic drugs^22–24^. In our previous study, we described the effect of surface functionalization of MSN on MTX loading and *in vitro* drug release, and demonstrated that thiol-functionalized MSN were suitable for MTX formulation, demonstrating a crystalline-to-amorphous transformation, high drug loading and pH-sensitive MTX release^25^.

Hypoxia and acidic extracellular conditions are hallmarks of tumor microenvironment. Hypoxia is an adaptive trait of progressive cancers and limited delivery of therapeutic agents to the hypoxic parts of solid tumors is recognized as one of the causes of resistance to chemotherapy. Efforts have been made to develop hypoxia-responsive therapeutics^26, 32^. Poon *et al*. successfully demonstrated selective localization of acidic pH-responsive, layer-by-layer nanoparticles in the hypoxic tumor microenvironment^27^. As hypoxia and subsequent acidosis are unifying factors for tumor cells to acquire resistance to chemotherapy and radiation, such targeted technologies may be helpful to sensitize tumor cells and decrease resistance^27, 28^.

Inspired by the pH-dependent release of MTX in our previous study, we hypothesized that MTX-MSN formulations will be more effective in hypoxic conditions when compared to normoxic conditions. Considering the inherent pH-dependent solubility and other physicochemical properties of MTX that promote increase in the cell uptake, we tested the effect of hypoxia and PEGylation on the properties of MTX-loaded MSN. For the first time, we report the application of PEGylated mesoporous silica nanorods (PMSNR) for delivery of anti-cancer drugs under hypoxic conditions. In this study, we demonstrate the effect of PEGylation of MSNR on MTX loading and we evaluate the *in vitro* release profile under hypoxic and normoxic conditions. We have loaded MTX into MSNR and PMSNR based on electrostatic adsorption. The effect of PEG on colloidal stability, hemolytic properties of MSNR, *in vitro* release of MTX, and cell killing efficiency under normoxic and hypoxic conditions were studied. PEGylation decreased the overall MTX loading but increased MTX release. It was also found that MTX-PMSNR and MSNR were more effective in the hypoxic than normoxic conditions.

## Materials and Methods

### Materials

Tetraethylorthosilicate (TEOS), 3-mercaptopropyltrimethoxysilane (MPTMS), *N*-cetyltrimethylammonium bromide (CTAB), Sodium hydroxide, hydrochloric acid, and sulfuric acid were purchased from Sigma-Aldrich. Mitoxantrone dihydrochloride (MTX) was purchased from Santa Cruz Biotechnology Inc. (Santa Cruz, CA). PEG-silane (MW 5000) was purchased from Laysan Bio Inc. Sheep whole blood (in sodium heparin) was purchased from Lampire Biological Laboratories. Roswell Park Memorial Institute medium (RPMI), phosphate buffered saline (PBS) (0.15 M, pH 7.4) and fetal bovine serum (FBS) were purchased from Invitrogen. Cell titer 96 Aqueous One solution cell proliferation assay (MTS reagent) was purchased from Promega.

### Synthesis of mesoporous silica nanorods (MSNR)

Thiol-functionalized MSNR were synthesized by co-condensation of TEOS and MPTMS using a modified surfactant-templated base catalyzed method which was reported previously^7, 10, 11, 29^. In a typical synthesis of SH-MSN, 1.0 g of CTAB was dissolved in 480 mL of deionized water made basic by the addition of 3.5 mL of 2.0 M NaOH, and the temperature was raised to 80 °C. To the rapidly stirred solution, 5.0 mL TEOS was injected at a rate of 1.0 mL/min using a syringe pump while stirring. The injection of TEOS was immediately followed by drop-wise addition of MPTMS (1.3 mmol), to achieve a molar ratio of TEOS: MPTMS of 8.7:1. The suspension was maintained at 80 °C for about 2 h and the final product was isolated by centrifugation. The isolated product was washed with excess deionized water and methanol and dried in vacuum. The removal of the CTAB template was carried out by refluxing the dried product in acidic methanol solution (18 mL of 12 M HCl, 20 mL of methanol) overnight. The particles were isolated by centrifugation, washed with methanol and de-ionized water, and dried overnight under active vacuum to yield a white powder.

### Characterization of MSNR

The morphology and size of the nanoparticles were characterized by transmission electron microscopy (TEM) on a JEOL 2010F Analytical Electron Microscope at 200 kV. TEM samples were prepared by placing a drop of a sonicated aqueous suspension of MSNR on a carbon-film copper grid. The surface area, average pore size, cumulative pore volume, and pore size distributions were determined from nitrogen adsorption/desorption isotherms acquired at 77 K using a 30 s equilibrium interval on an ASAP 2010 Micromeritics porosimeter or a TriSTAR II porosimeter. The surface area was computed using the Brunauer-Emmett-Teller (BET) model. The cumulative pore volume was obtained from the BJH (Barret-Joyner-Halenda) model and the pore size distribution was obtained from density functional theory (DFT) modeling using the DFT package of the Micromeritics V2.00 software over the entire range of the adsorption isotherm.

### PEGylation of MSNR

Grafting of PEG-silane on the MSNR surface was achieved by using a modified method reported previously^17^. The MSNR surface was modified with an increasing amount of PEG-silane with the overall PEG-to-MSNR w/w ratios of 0.2, 0.4, 1 and 5. In a typical experiment, 50 mg of MSNR were suspended in 2 mL of anhydrous toluene followed by sonication for 2–3 min. The resultant MSNR suspension was heated to 110 °C and a PEG-silane solution in 4 mL anhydrous toluene was added dropwise to the stirred MSNR suspension. Particles were stirred for 12 h and isolated by centrifugation at 13,300 rpm for 5 min, followed by washing with ethanol to remove the unreacted PEG-silane. PEGylated MSNR (PMSNR) were dispersed in 2 mL of DI water and lyophilized to obtain a free flowing powder. PMSNR were analyzed by thermogravimetric analysis (TGA) for PEG content (Perkin-Elmer Pyris 1, 10 °C/min).

### Colloidal stability

Colloidal stability was characterized by dynamic light scattering (DLS). PMSNR or MSNR (1 mg) was dispersed in 1 mL of RPMI containing 10% FBS followed by analysis using a Zeta Plus particle size analyzer (Brookhaven Instrument) for 5 h. The intensity of the scattered light (kcps) at 90° was measured at the same time and plotted against time to evaluate aggregation and sedimentation.

### Hemolysis assay

Hemolytic properties of MSNR and PMSNR were determined by a previously reported method^30^. In a typical experiment, 2 mL of whole sheep blood was centrifuged at 3,000 rpm for 10 min and the supernatant containing plasma and white blood cells was discarded. The red blood cells (RBC) were washed with PBS multiple times until the supernatant became colorless. The hemolysis assay was performed in triplicate in a 96-well microplate and 120 μL of the RBC suspension was added to each well. 1% triton X-100 was used as a positive control and PBS was the negative control. Increasing concentration of MSNR and PMSNR in PBS was added to make the final volume to 150 μL, followed by incubation at 37 °C for 1 hr. The plate was then centrifuged at 3,800 rpm for 5 min and 20 µL of the supernatant in each well was further diluted to 120 μL before measuring absorbance at 414 nm to determine hemoglobin release. The positive control, 1% triton X-100, was set to 100% hemolysis. The results are expressed as mean ± S.D. (n = 3).

### MTX loading

In a typical experiment, MTX was dissolved in PBS at a concentration of 2 mg/mL. A calculated amount of MTX solution was added to 1 mg of dry MSNR or PMSNR particles at various w/w ratios. The mixture was then sonicated for 30 min and stirred for another 24 h. The drug-loaded particles were centrifuged at 14,500 rpm for 10 min and vacuum dried overnight. The MTX concentration in the supernatant (non-loaded MTX) was determined from absorbance at 658 nm based on MTX standard curve. The amount of MTX loaded in particles was calculated by subtracting the non-loaded MTX from the original MTX solution. Drug loading (weight %) was calculated as:

$$Drug\,loading\, \% =\frac{weight\,of\,loaded\,MTX}{weight\,of\,MTX\,loaded\,particles}\times 100$$

### Hydrodynamic radius and ζ potential measurement

The measurement of hydrodynamic radius and ζ potential of MSNR and PMSNR (+/−MTX loading) was performed by DLS using a ZetaPlus Particle Size and Zeta Potential Analyzer (Brookhaven Instruments) equipped with a 35 mW solid state red laser. Scattered light was detected at 90° and the temperature was set at 25 °C. Samples were prepared by suspending 200 μg of particles in PBS at a concentration of 100 μg/mL. The mean hydrodynamic radius was calculated for size distribution by weight, assuming a lognormal distribution using the supplied algorithm and the results are expressed as mean ± S.D. of five runs.

### MTX Release

The release of MTX from MSNR and PMSNR was analyzed by suspending a known amount of the MTX-loaded particles in the release medium (PBS at pH 7.4, or 0.2 M sodium acetate buffer at pH 4.5). At each pre-determined time point, particles were centrifuged down at 14500 rpm for 10 min. A sample of the supernatant (1 mL) was taken and replaced with 1 mL of fresh release medium to maintain the sink conditions. MTX concentration in the sampled supernatant was determined by measuring absorbance at 658 nm using a pre-constructed standard curve in the corresponding release buffer. The results are expressed as mean ± S.D. (n = 3).

### Cell Culture

The triple negative human breast cancer cell line MDA-MB-231 was a kind gift from Dr. Jing Li, Barbara Ann Karmanos Cancer Institute (Detroit MI). The cells were cultured in Hyclone’s RPMI medium supplemented with L-glutamine, 10% FBS and 1% penicillin. For general culturing in normoxic condition, the cells were maintained in an incubator at 37 °C with 5% CO_2_. To achieve hypoxic condition, the cells were maintained in an incubator installed with oxygen controller (ProOx model 110, BioSpherix) at 37 °C with 5% CO_2_ and 5% oxygen.

### Cell Viability

Cytotoxicity of drug-loaded MSNR and PMSNR was determined by the CellTiter 96® Aqueous Cell Proliferation (MTS) Assay. MDA-MB-231 cells cultured in either normoxic or hypoxic condition were seeded in a 96-well plate at a density of 5,000 cells per well one day before any type of treatment. MTX loaded particles with increasing MTX concentrations in 100 μL culture medium were added and incubated for 72 h. After the incubation, the medium was removed and replaced with a mixture of 100 μL serum-free RPMI and 20 μL MTS reagent solution. The absorbance of each well was then measured at 490 nm to determine cell viability after 1 h incubation. The results are expressed as mean % cell viability relative to the untreated cells ± S.D. IC50 values were determined by Prism software using non-linear regression involving log (inhibitor) vs. response (three parameters) analysis of dose-response inhibition. The results are represented as mean ± S.D. (n = 3).

### Cell uptake

The cell uptake of MTX-loaded MSNR and PMSNR was determined by measuring cell-associated fluorescence of MTX using flow cytometry. MDA-MB-231 cells cultured in normoxic or hypoxic conditions were seeded in a 24-well plate at a density of 2.5 × 10^5^ cells per well 12 h prior to the experiment. Cells were incubated with pre-determined concentration of free MTX, MTX-loaded MSNR and PMSNR in the culture medium under normoxic or hypoxic environment for 2 h. Cells were then washed twice with PBS, and harvested after trypsinization. Cells were resuspended in Hank’s buffered salt solution (HBSS) and the mean fluorescent intensity was analyzed by flow cytometry (Ex 658 nm/Em 670 nm). The flow cytometry analysis was performed on a BD Biosciences LSR II instrument, and 10,000 cells were collected for each measurement. Cellquest software was used for data analysis. Reported fluorescence intensity data were corrected for cell autofluorescence using untreated cells.

## Results

### Synthesis and characterization of PMSNR

**Figure 1:**
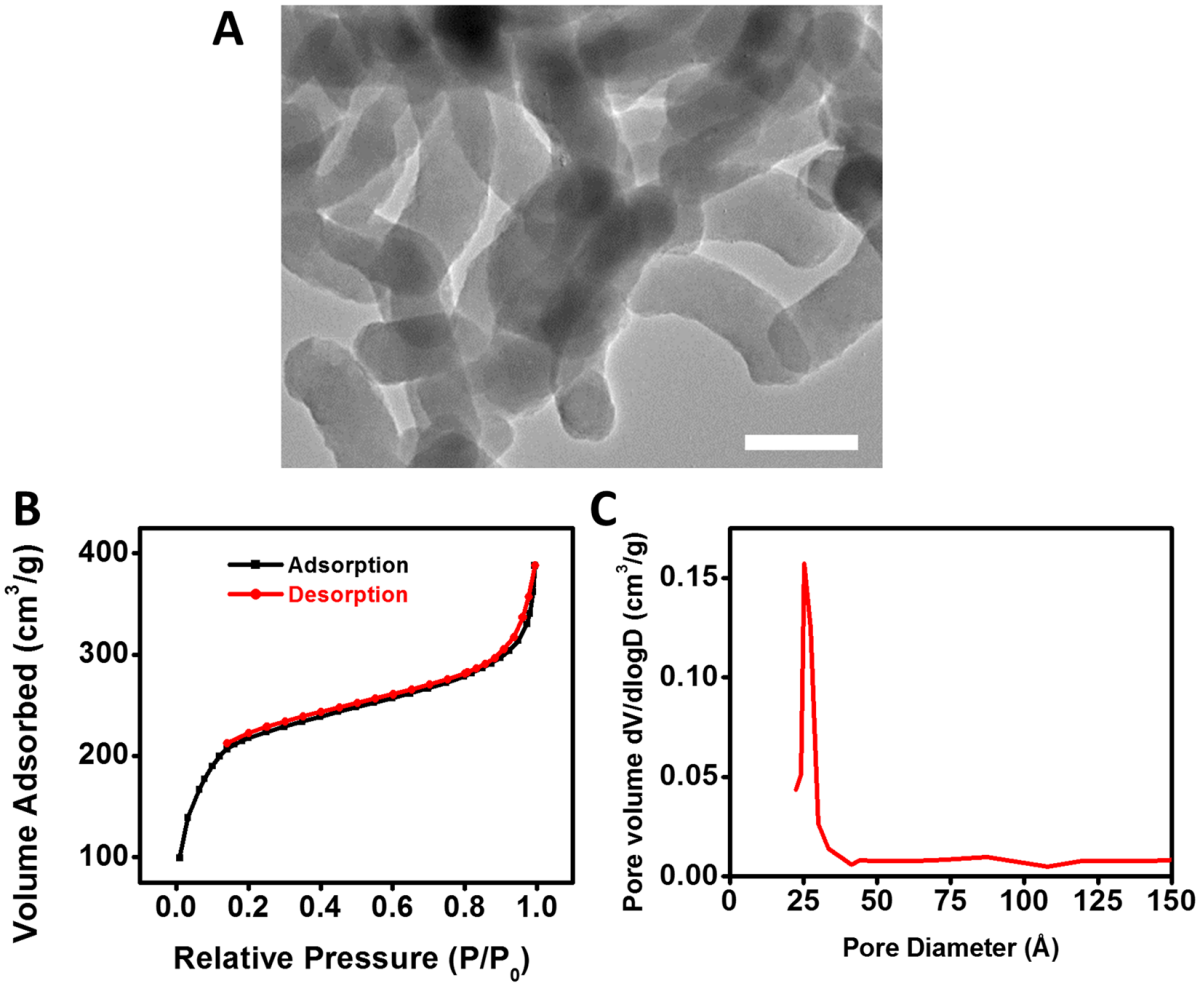
Physiochemical characterization of MSNR. (**A**) TEM image of MSNR (Scale bar = 100 nm). (**B**) Type IV adsorption isotherm measured by nitrogen physisorption and (**C**) Pore size distribution of MSNR.

**Figure 2:**
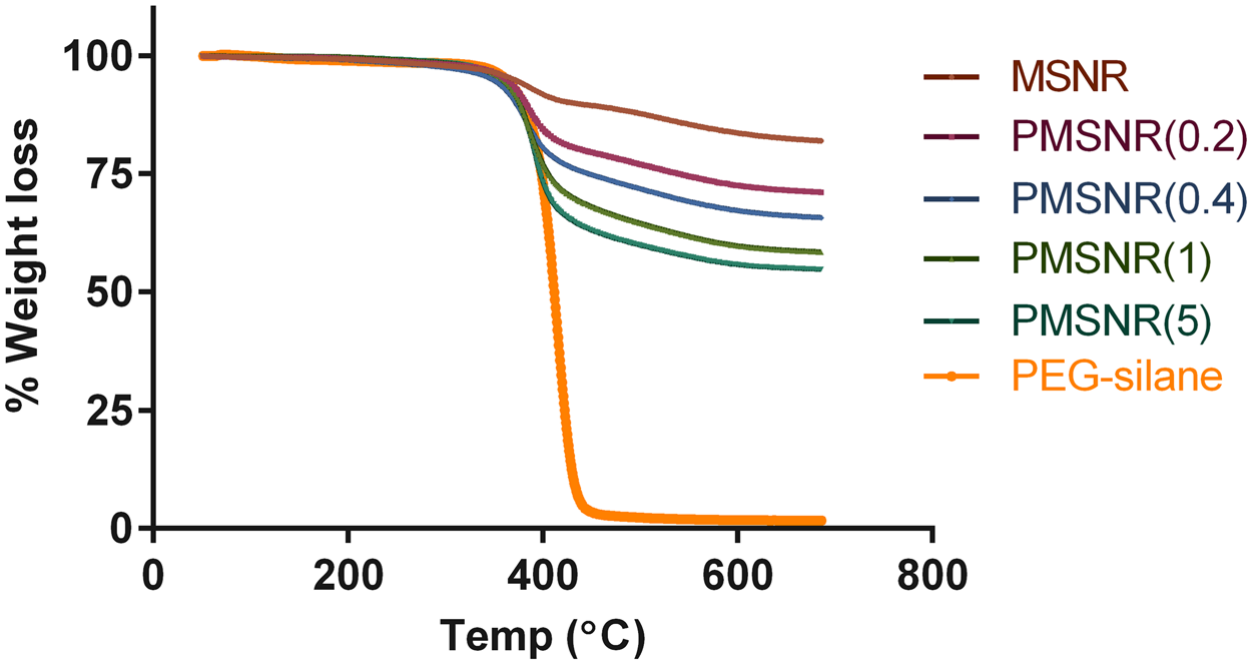
Thermogravimetric analysis (TGA) of MSNR and PMSNR. TGA was performed in air with the temperature ramped from 50–700 °C at a rate of 1 °C/min.

MSNR were synthesized using a surfactant-templated base-catalyzed method and a representative TEM image is shown in Fig. 1A. MSNR exhibited a rod-like shape with overall dimensions of 120 nm × 25 nm (length × width). The nitrogen adsorption isotherm (Fig. 1B) revealed that MSNR followed a type IV adsorption isotherm, which is typical of an MCM-41 type pore structure. The total surface area of the particles was determined to be 820 m^2^g^−1^. BJH analysis revealed a narrow pore size distribution with an average pore size of 2.6 nm (Fig. 1C). Various PMSNR with different PEG-to-MSNR w/w ratios were synthesized. Successful PEGylation of MSNR surface was confirmed by TGA analysis. As shown in Fig. 2, increasing the feed amount of PEG resulted in increased content of PEG in the final product. The synthesized PMSNR had a final PEG weight content ranging from 15 to 33.2% (Table 1).

**Table 1:** PEGylation of PMSNR.

MSNR:PEG (w/w) in feed	PEG content in PMSNR by TGA (wt %)
1:0.2	15
1:0.4	22.3
1:1	29.5
1:5	33.2

**Figure 3:**
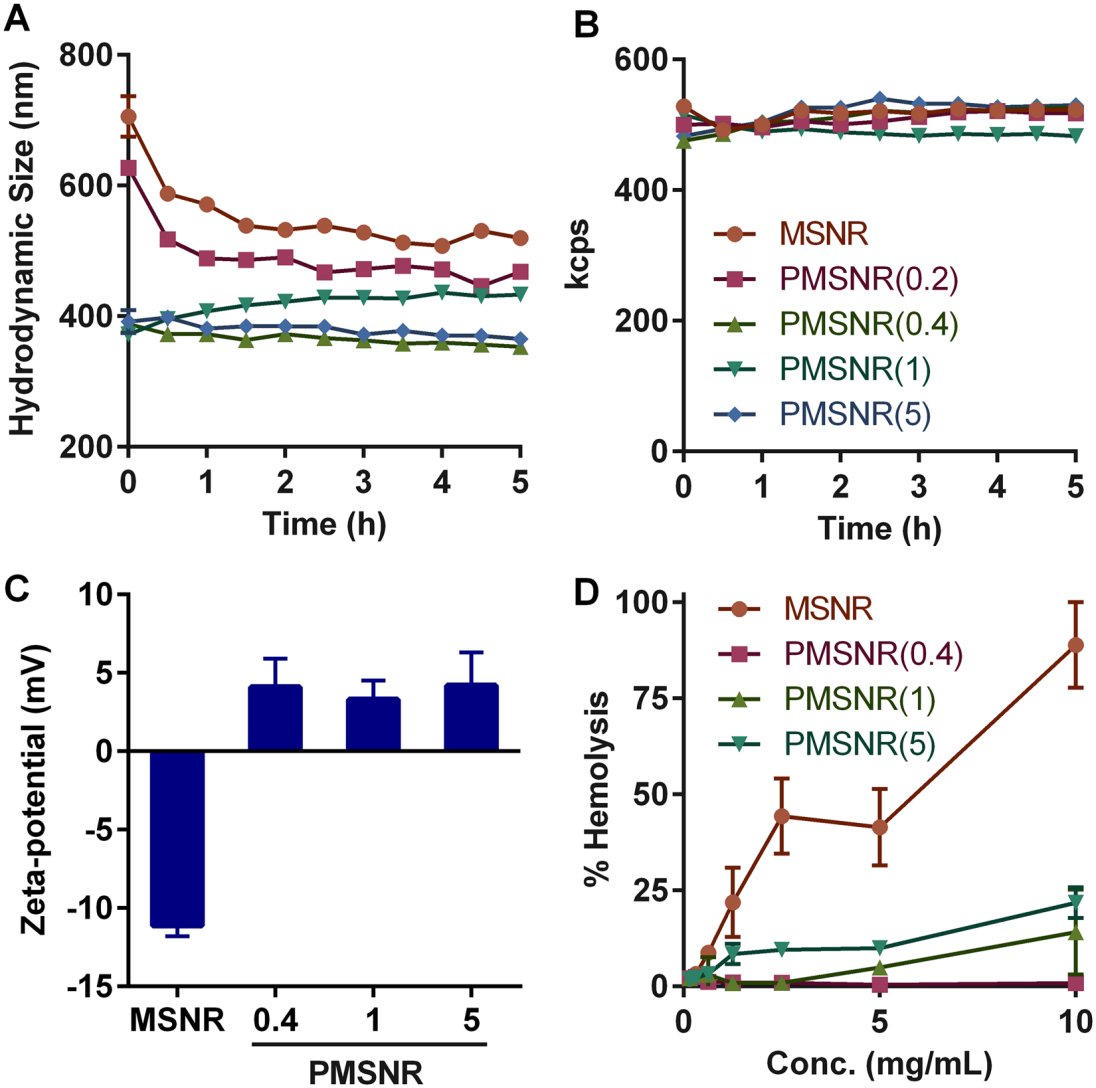
Effect of PEGylation on (**A**,**B**) colloidal stability, (**C**) zeta potential, and (**D**) hemolysis of MSNR. Colloidal stability was determined by measuring particle size and scattering intensity (kcps) in RPMI medium containing 10% FBS for 5 h. Hemolysis was analyzed by incubating MSNR with sheep RBCs for 2 h.

Following successful synthesis, the effect of PEGylation on the colloidal stability of MSNR was characterized (Fig. 3). We first measured hydrodynamic size of the particles in the RPMI cell culture medium in the presence of 10% FBS and found that PEGylation resulted in significantly decreased size (0 h time point in Fig. 3A). The colloidal stability was then examined by following changes in particle size over time. As shown in Fig. 3A, PEGylation significantly improved the colloidal stability of MSNR. In the serum-containing medium, the parent MSNR exhibited an initial large particle size around 700 nm as a result of extensive interactions with serum proteins and associated particle aggregation. The size of MSNR decreased until a plateau was achieved with a stabilized size of 520 nm. PMSNR with high PEG content (PEG/MSNR w/w ≥ 0.4) exhibited a sterically stabilized particle size around 400 nm over the course of the 5 h incubation and these particles were then used in all subsequent studies. Despite the interactions with serum proteins, no sedimentation of the particles was observed, which was indicated by the unchanging intensity of the scattered light (Fig. 3B).

The effect of PEGylation on the surface charge of MSNR was explored by measuring ζ potential of the particles in sodium phosphate buffer (20 mM, pH 7.4). As shown in Fig. 3C, a clear shift in the ζ-potential from negative to slightly positive was observed after PEGylation. MSNR displayed a negative surface charge of −11.1 ± 0.7 mV, while all the PMSNR particles were nearly neutral, which further confirmed the successful PEGylation and shielding of the MSNR surface.

Hemolytic properties of MSNR and PMSNR were determined using sheep RBCs, and 1% triton X-100 was used a positive control (100% hemolysis) (Fig. 3D). The results indicate that MSNR caused substantial hemolysis of the RBCs and that the hemolysis increased in a dose-dependent manner within the tested dose rage (0–10 mg/mL). In contrast, PMSNR showed significantly reduced membrane damage and lysis of RBCs. PMSNR (0.4) in particular, exhibited only marginal (<1%) hemolysis even at the highest concentrations tested. Interestingly, PMSNR with higher PEG content (PEG/MSNR w/w 1 and 5) showed higher hemolytic activity than PMSNR (0.4) suggesting the need for careful optimization of the PEG content in the particles.

### MTX Loading

**Figure 4:**
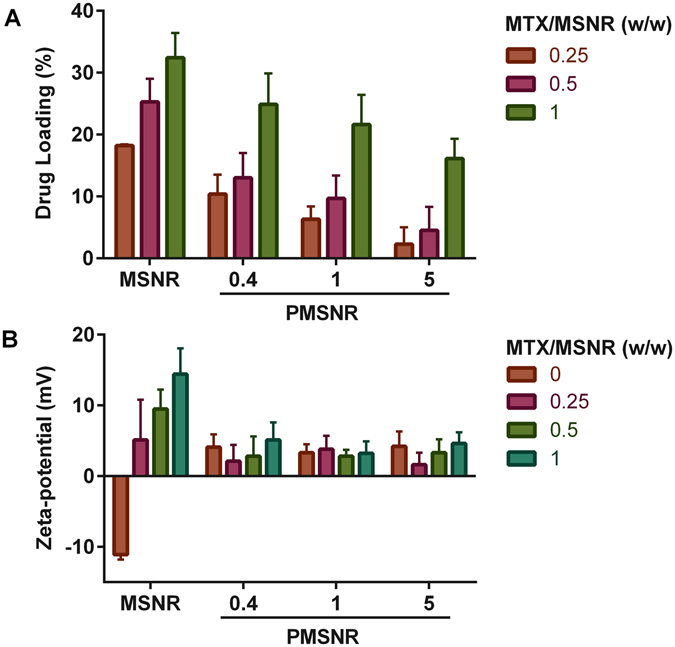
Effect of PEGylation of MSNR on (**A**) MTX loading, and (**B**) zeta-potential. The MTX loading results are presented as mean ± S.D. (n = 3). Zeta-potential was measured in sodium phosphate buffer (20 mM, pH 7.4), and the results are presented as mean ± S.D. (n = 5).

The loading mechanism of MTX in MSNR relies on the electrostatic interactions between the cationic MTX and the anionic MSNR at the physiologic pH. Here, we investigated the effect of PEGylation on the loading of MTX in MSNR by using increasing concentrations of MTX in the loading solution. Figure 4A shows that the MTX loading in MSNR increased from 18% to 34% when MTX/MSNR w/w ratio in the loading solution increased from 0.25 to 1. A similar trend was observed in all the PMSNR, however the overall MTX loading decreased with increasing PEG content. This suggests that MTX loading is hindered by the presence of PEG on the MSNR surface. We have also studied the effect of MTX loading on the ζ potential (Fig. 4B). A charge reversal from negative to positive was observed in the ζ-potential of MSNR after MTX loading, and the surface charge of the particles increased with increasing loading of MTX. However, in the case of PMSNR, only a negligible change in the ζ potential was observed after MTX loading, confirming the beneficial surface shielding effect of PEG.

### MTX Release

**Figure 5:**
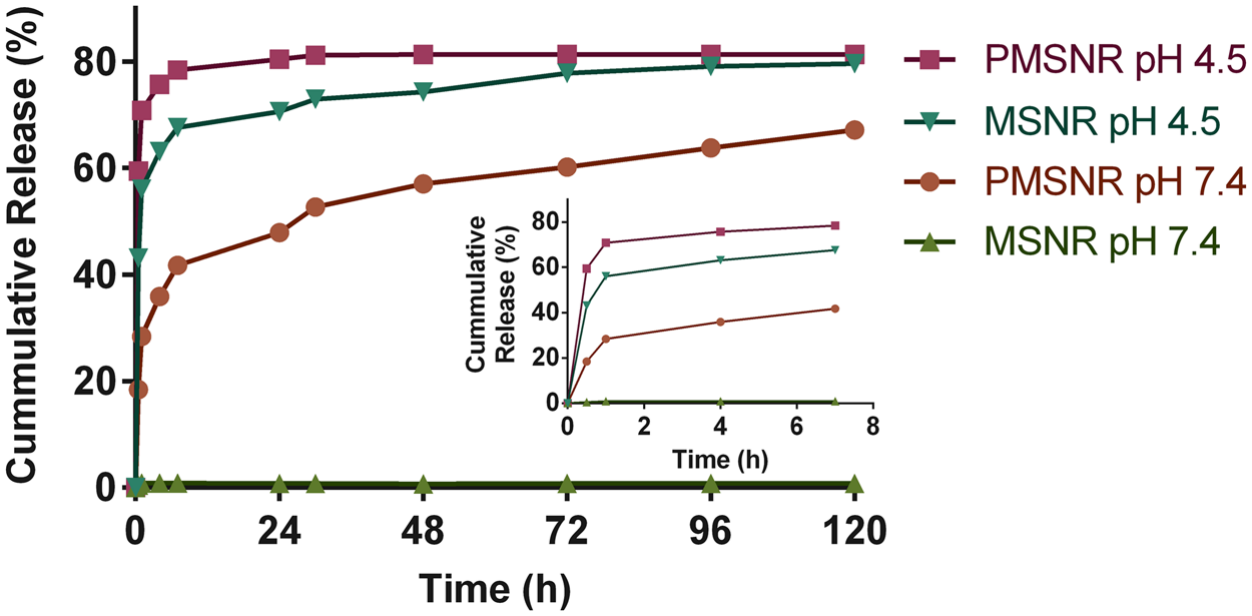
Effect of PEGylation of MSNR on drug release. The released amount of MTX was determined from supernatant absorption at 658 nm. (Inset: detailed release within the first 7 h).

Based on the above results, PMSNR (0.4) emerged as the best performing particles with stable size in simulated physiologic conditions, near-neutral surface charge, low hemolytic activity, and high MTX loading. We then investigated the drug release profile in both PBS (0.15 M, pH 7.4), which mimics the physiological conditions, and acetate buffer (0.2 M, pH 4.5) used to mimic intracellular conditions during endo/lysosomal trafficking of the nanoparticles. As shown in Fig. 5, the release of MTX from MSNR was highly pH-dependent. MSNR released 56% of MTX within the first hour at pH 4.5, while only marginal release was observed at pH 7.4 even after 120 h (<1%). The MTX release form PMSNR also revealed pH dependence; however it was not as pronounced as in the case of MSNR. PMSNR showed sustained release of MTX at pH 7.4 while exhibiting faster release of MTX at pH 4.5 (71% within the first hour) than MSNR.

### Cytotoxic Activity in Hypoxic and Normoxic Conditions

**Figure 6:**
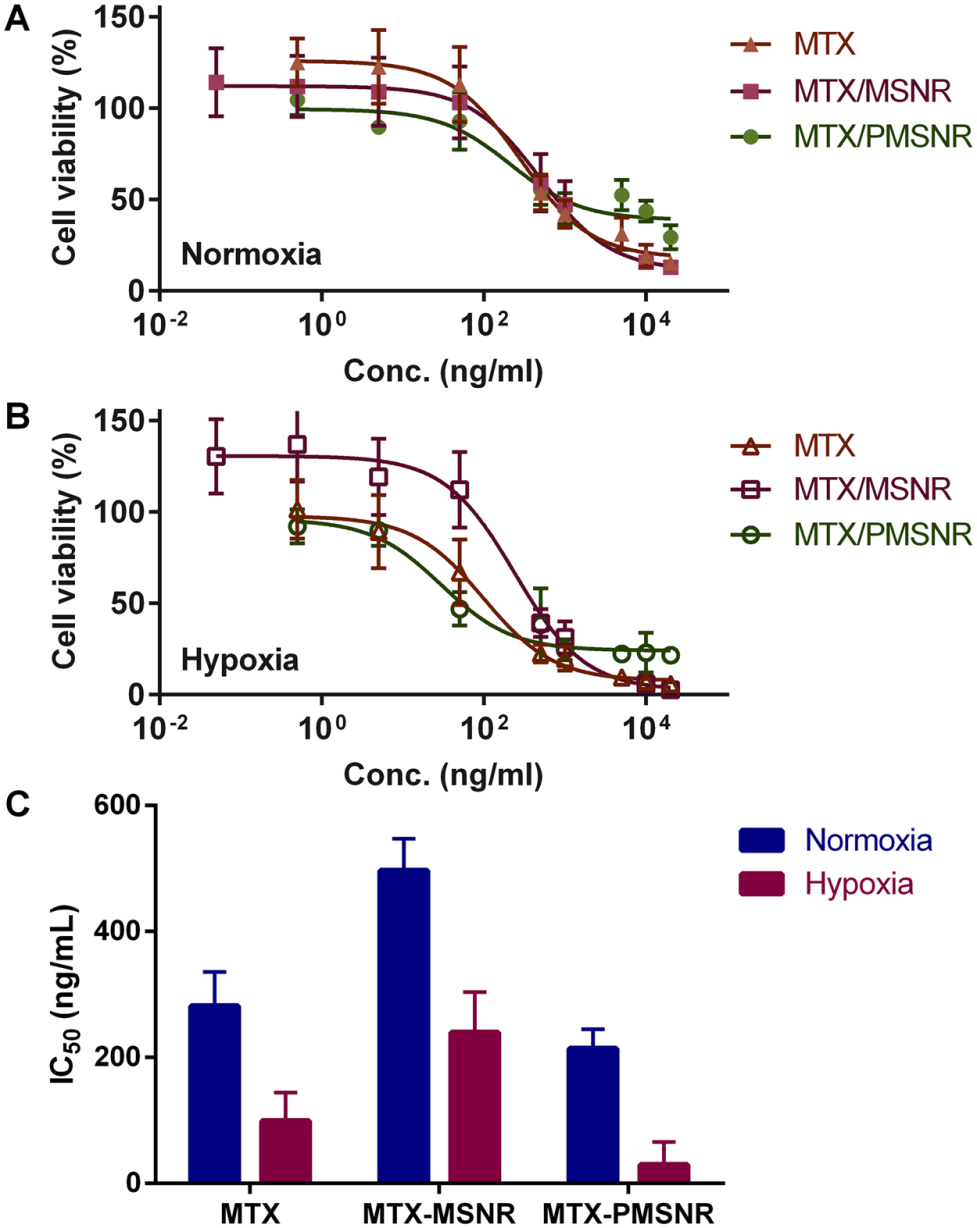
Cytotoxicity of MTX-loaded MSNR and PMSNR in MDA-MB-231 cells under (**A**) normoxia and (**B**) hypoxia. IC50 values are summarized in (**C**).

The cytotoxic activity of MTX-loaded MSNR and PMSNR under hypoxic and normoxic conditions was evaluated in human triple-negative breast cancer cell line MDA-MB-231. Free MTX was used as the control. Free silica nanoparticles showed no significant cytotoxicity under the used experimental conditions. Dose-response curves for MTX, MTX-loaded particles in normoxia (Fig. 6A) and hypoxia (Fig. 6B) were constructed and the corresponding IC_50_ values were calculated and are summarized in Fig. 6C. The results show that the IC_50_ for free MTX under normoxic conditions was 281.9 ± 53.9 ng/mL. Encapsulation of MTX in MSNR decreased the drug potency as suggested by a higher IC_50_ of 496.7 ± 50.5 ng/mL. In contrast, loading MTX in PMSNR resulted in significantly higher cell killing activity (IC_50_ of 214.3 ± 30.3 ng/mL) when compared with both MSNR and even with free MTX. In hypoxic conditions, all three formulations showed marked decrease in IC_50_, suggesting enhanced cytotoxic effect. PMSNR in particular, demonstrated improved anticancer activity with the lowest estimated IC_50_ of 29.9 ± 35.9 ng/mL among all the tested groups. MTX and MTX/MSNR showed 3.3- and 8-fold increase in the IC_50_ value, respectively.

### Cell Uptake in Hypoxic and Normoxic Conditions

**Figure 7:**
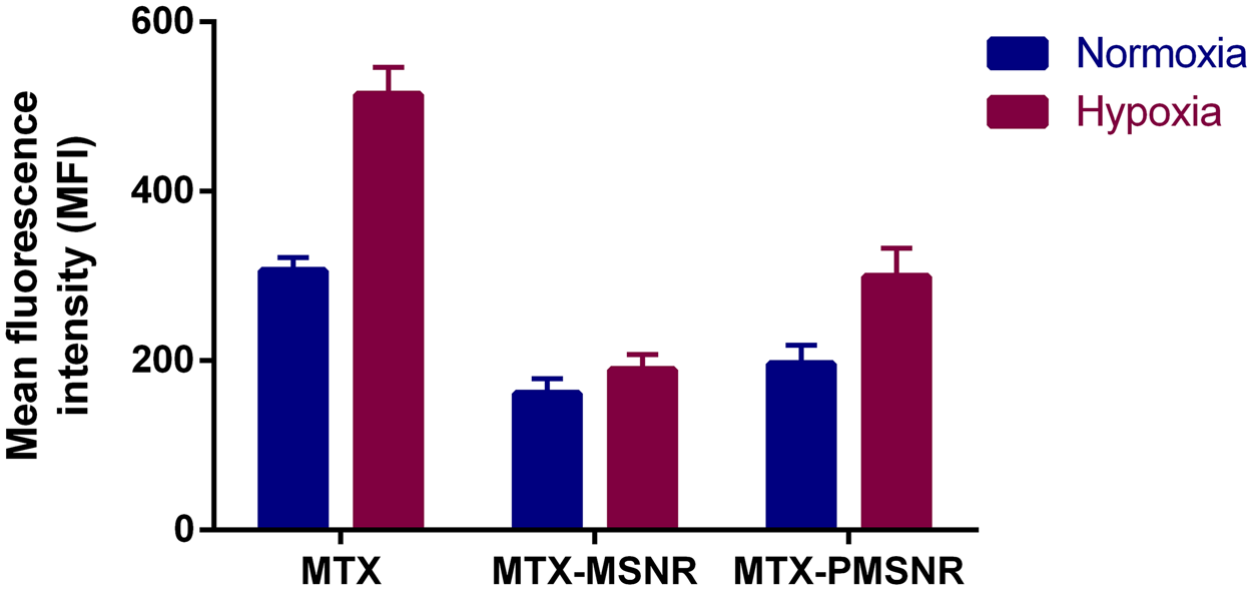
Effect of hypoxia on MDA-MB-231 cell uptake of MTX and MTX-loaded MSNR and PMSNR. Cell uptake shown as mean fluorescence intensity (MFI) of free MTX in the cells measured by flow cytometry.

The initial cell uptake and intracellular release of MTX-loaded MSNR and PMSNR under hypoxic and normoxic conditions were further evaluated in MDA-MB-231 cells. As shown in Fig. 7, in both conditions, free MTX showed higher uptake than either MTX-loaded MSNR or PMSNR particles. In this experiment, only free intracellular MTX was measured by FACS due to quenching of MTX fluorescence when loaded in the nanoparticles^22^. MTX loaded in MSNR exhibited similar cell uptake in both normoxic and hypoxic conditions (mean fluorescence intensity per cell (MFI) = 161 vs. 189), while when the drug was loaded in PMSNR, the uptake was significantly higher (~1.53 fold) in hypoxia than in normoxia (MFI 299 vs. 196).

## Discussion

Due to its outstanding control over particle size and shape, MSN are emerging as an attractive material for a wide range of applications from drug delivery to theranostics^31, 32^. To achieve controlled drug release, versatile approaches such as PEGylation, the use of nanovalves^33^, pH-sensitive polymer shells^34^ and various surface polymer modifications^13, 35^ have been explored and applied to the design of MSN-based delivery systems. PEGylation of nanoparticles is widely used to stabilize the particles, modify their renal clearance to improve biodistribution, prolong the circulation time and prevent opsonization by reducing the overall surface charge^18, 21, 36^. However, PEGylation also has a dramatic impact on the surface properties that may affect the drug loading and release. We previously reported that surface modification of MSN with poly(2-(dimethylamino)ethylmethacrylate) or poly(2-(diethylamino)ethylmethacrylate) leads to synergistic delivery of chloroquine and nucleic acids in cancer cells *in vitro*. PEGylation of those particles, however, resulted in a decrease in chloroquine loading from 73% to 43%^8^. Singh *et al*. reported a MSN system for drug and gene delivery application and they also observed a drop of doxorubicin loading from 40% to 3% after PEGylation^12, 13^. Such a decrease in drug loading due to PEGylation inspired us to fill the gap of knowledge in how PEGylation of MSN affects the physicochemical characteristics and biological activity of the particles and how such knowledge can be utilized in optimizing PEGylation of MSN to retain acceptable levels of drug loading and release, while exploring the effect of hypoxia on activity of MTX-loaded PEGyated MSN.

In this study, we focused on rod-shaped MSN and explored the effect of surface PEGylation on the loading and release kinetics of MTX, an anticancer small-molecule drug. Particle shape is known to play a critical role in controlling the therapeutic outcomes of the delivery systems due to a change in the rate of intracellular uptake^6^, effect on the blood circulation time^7^, and the extent of particle opsonization^37^. MTX is a weakly basic drug with two types of secondary amines with p*K*_a_ = 5.99 and 8.13. Unmodified MSNR exhibits negative surface charge due to the silanol surface groups. Thus, the MTX loading in MSNR relies on electrostatic interactions between the two entities and the drug loading is strongly influenced by the surface charge of the particles as well as the pH of the loading solution. Our results showed that loading of MTX into MSNR could reverse the particle surface charge from negative to positive, as indicated by Fig. 4B. The surface charge was also highly dependent on the w/w ratio of MTX and MSNR. Increasing the MTX content in the loading solution not only resulted in higher ζ potential of the particles, but also led to higher drug loading (Fig. 4A). The highest MTX loading in MSNR we achieved was 34%. After surface PEGylation, the ζ potential of the particles became nearly neutral (Fig. 3C), which adversely affected the loading capacity for the positively charged MTX. We tested a series of PMSNR with different PEG content, and the results showed that PMSNR(0.4) showed the least effect on drug loading (Fig. 4A), while providing acceptable colloidal stability in serum-containing medium (Fig. 3A).

Interactions with RBCs are a major concern in the development of any nanoparticles intended for systemic administration. Importantly, unmodified MSN are already known for their high hemolytic activity^15, 17^. It has been shown that hemolytic activity of MSN is highly correlated with the particle size, total surface area and the number of surface silanol groups^15, 17^. PEGylation is a viable and effective approach to counter the hemolytic nature of MSN^15, 38^. Our results confirmed that all the PMSNR reduced the hemolysis significantly since PEGylation alters the surface charge of the particles and further reduces the interactions with RBCs, leading to enhanced blood biocompatibility (Fig. 3D). An interesting observation is that among all the PMSNR tested, it was PMSNR (0.4) that exhibited the lowest hemolytic activity and not the PMSNR with higher PEG content. This is most likely related to the previously reported membranolytic activity of PEG when present at high local concentrations such as those found on the surface of PEGylated nanoparticles.

*In vitro* drug release from MSN has been well studied with respect to varying pore size^39^, the functional groups on the walls of the pores^40^, drug loading and the choice of loaded therapeutic agent^16^. Our results showed that the MTX release is strongly dependent on pH. In MTX-loaded MSNR, marginal release was detected at neutral pH and significantly faster release was found in acidic environment. Such pH dependence suggested the capability of MSNR to achieve controlled release of MTX in treating hypoxic tumors, as the drug molecules remain in the particles during *in vivo* circulation while being released at the acidic and hypoxic tumor microenvironment. Interestingly, PEGylated MSNR demonstrated a distinct drug release profile. Although pH dependency still existed in the case of PMSNR, it was not as pronounced as in MTX-loaded MSNR. A significant increase in the rate of MTX release of PMSNR was observed at neutral pH compared with MSNR. Such difference is most likely due to decrease in the strength of the electrostatic interactions between MTX and the silica matrix caused by PEGylation, especially on the surface of the particles.

*In vitro* assessment of anti-cancer agents in hypoxic conditions promises to increase the significance of such findings compared when the experiments are performed in normoxic conditions. This is because hypoxia (and related acidosis) is a typical feature of solid tumors. Hypoxic tumors are usually associated with elevated production of hypoxia-inducible factor (HIF-1), which plays an important role in the development of multidrug resistance. MTX has been shown to inhibit preferentially HIF-1α under hypoxic conditions^41^. MTX could successfully inhibit HIF-1α expression and accumulation in hypoxic tumors. We thus evaluated the cell cytotoxic activity of MTX-loaded MSNR and PMSNR in hypoxic vs. normoxic conditions in MDA-MB-231 breast cancer cells. As expected, free MTX exhibited significantly enhanced cell killing effect in hypoxia than in normoxia. Importantly, such activity enhancement in hypoxia was also observed in both MTX-loaded particles. Interestingly, the order of cytotoxic activity of MTX in both hypoxic and normoxic conditions was as follows: MTX-PMSNR > free MTX > MTX-MSNR. In other words, MTX-loaded PMSNR exhibited the highest cell-killing activity, which could be partially attributed to the favorably fast drug release following internalization into the cancer cells (Fig. 5).

The enhanced cytotoxic activity of MTX-PMSNR over MTX-MSNR could also be related to the higher cellular uptake. To test this hypothesis, we evaluated the cell uptake of MTX-loaded MSNR and PMSNR in the MDA-MB-231 cells. As the fluorescent signal from MTX is quenched when loaded inside the particles, the cell uptake results indicate the amount of released/free MTX located inside of the cells. As shown in Fig. 7, significantly higher delivery of free MTX to the cancer cells was observed in all the formulations under hypoxia than in normoxia. Free MTX exhibited the highest cell uptake in both conditions as the uptake mostly relies on passive diffusion^42^. MTX-loaded MSNR and PMSNR exhibited lower cell uptake at 2 h, which was expected considering the different uptake mechanism of the particles and the fact that it takes time for the drugs to escape from the pores of the particles. Despite the well-established phenomenon of compromised cell uptake of PEGylated nanoparticles^43^, we have observed higher amount of MTX found in the cells when delivered by PMSNR compared to non-PEGylated MSNR. This agrees well with the results of the MTX release data MTX as PMSNR showed a faster release of MTX in both neutral and acidic pH (Fig. 5).

## Conclusion

We have successfully demonstrated the effect of PEGylation of MSNR on loading and release of MTX. PEGylation of MSNR decreased overall drug loading but increased the rate of MTX release. PEGylation of MSNR minimized the extent of hemolysis. Cytotoxicity studies showed that the MSN MTX formulations were as effective as free MTX in hypoxic conditions but less effective in normoxic conditions. We conclude that PMSNR represent a promising system for MTX delivery but further optimization is necessary to develop them as injectable formulations.
